# *Coxiella burnetii* Detected in Tick Samples from Pastoral Communities in Kenya

**DOI:** 10.1155/2018/8158102

**Published:** 2018-07-09

**Authors:** Hellen Koka, Rosemary Sang, Helen Lydia Kutima, Lillian Musila

**Affiliations:** ^1^Jomo Kenyatta University of Agriculture and Technology, P.O. Box 62000-00200, Nairobi, Kenya; ^2^US Army Medical Research Directorate-Kenya, P.O. Box 606-00621, Nairobi, Kenya; ^3^Kenya Medical Research Institute, Centre for Virus Research, P.O. Box 54628-00200, Nairobi, Kenya; ^4^International Centre for Insect Physiology and Ecology, P.O. Box 30772-00100, Kenya

## Abstract

Ticks are important disease vectors in Kenya with documented evidence of carriage of zoonotic pathogens.* Coxiella burnetii *is an important tick-borne pathogen that is underreported in Kenya and yet this infection likely contributes to undiagnosed febrile disease in pastoral communities. Archived human blood (278) and tick pool samples (380) collected from five pastoral communities in Kenya were screened for* C. burnetii* by PCR using primers targeting the transposon-like IS1111 region. All the human blood samples were negative for* C. burnetii* DNA. However,* C. burnetii* was detected in 5.53% (21/380) of the tick pools tested. Four of the twenty-one PCR positive samples were sequenced. The findings indicate that* Coxiella burnetii* was not present in the human blood samples tested. However,* C. burnetii* was detected in ticks from Mai Mahiu, Marigat, Ijara, Isiolo, and Garissa indicating a natural infection present in the tick vector that poses a risk to livestock and humans in these communities.

## 1. Introduction

Q fever, a zoonotic disease caused by* Coxiella burnetii*, has emerged as an important human and veterinary public health problem worldwide [1]. In humans, Q fever may be asymptomatic or manifest as an acute febrile illness with pneumonia [2]. In a small number of patients, the disease may progress to a chronic form that is mainly associated with patients who are immunocompromised [3]. It may also be seen in patients who have preexisting heart valve defects presenting as endocarditis [4, 5].

Domestic animals such as cattle, sheep, and goats are the primary reservoirs of* C. burnetii *[6]. In animals, infections are mainly asymptomatic but still births, late abortion, delivery of weak offspring, and infertility are reported to occur [7]. The infected animal sheds the bacterium through the placenta and birth fluids which may contaminate the environment. Contamination of the environment leads to airborne dissemination of the bacterium and infection of persons in close contact with livestock [8–10]. As a result, Q fever is often an occupational disease affecting farmers, veterinarians, and abattoir workers [5].

Although transmission to humans and susceptible animals occurs primarily through inhalation of* C. burnetii *spores from the environment, humans may also get infected through contact with milk, urine, faeces, vaginal mucus, or semen of infected animals [11]. Cases of Q fever in man are mainly precipitated by infection in animals [12] yet, an outbreak of Q fever in humans is what leads to the investigation of livestock in most countries [9]. In fact, an outbreak in the human population can be prevented by monitoring domestic animals for signs of abortion or birthing of weak offspring which indicate possibility of Q fever infection [13].

Ticks are considered the natural primary vector of* C. burnetii* as they maintain the infection in domestic animals [5]. Transmission of* C. burnetii* is by tick bite or exposure to infected excreta expelled by ticks onto the skin of the animal host as they feed [4, 7]. In experimental studies, ticks readily transmit* C. burnetii* to humans [14]. However, direct transmission to humans from an infected tick is not well documented and may occur only rarely in nature [15]. The main transmission route from ticks to humans is therefore considered to be via inhalation of contaminated fecal material from ticks [1, 4].

Outbreaks of Q fever in man and domestic animals have been reported in many European countries [1, 16, 17] but since Q fever is not routinely tested for, it is likely underreported in Africa [12]. Since there is a growing interest in the role of nonmalarial causes of fevers [2], documented reports of Q fever infections are increasing as more diagnostic tools become available [18]. For instance, a study in Tanzania demonstrated that Q fever was the cause of 5% of febrile illness in hospitalized adult and paediatric patients [19]. Another study in Namibia, evaluating the causes of febrile illness, reported a 26% seroprevalence of Q fever in blood donors [20]. Furthermore, a study in Chad evaluating zoonotic diseases in three nomadic communities reported a 1% prevalence rate of Q fever [21].

In Kenya, the prevalence of Q fever was reported at 12.1% in both livestock and human populations in five of seven provinces [22]. The North Eastern and upper Eastern regions of Kenya which have large nomadic pastoral communities were not included in the sero-survey, yet they keep large herds of livestock [23] and the risk of Q fever transmission is reportedly higher in grazed animals [11]. Although it is clear that livestock husbandry systems play a key role in the transmission of Q fever to humans, infection rates in nomadic communities in Kenya have not been determined. The fact that Q fever is reportedly higher in grazed animals and that nomadic pastoral communities keep large herds of livestock, a study to determine the prevalence of Q fever (*C. burnetii)* in human and tick samples from several pastoral communities in Kenya was carried out.

## 2. Materials and Methods

### 2.1. Ethical Approval

**Figure 1:**
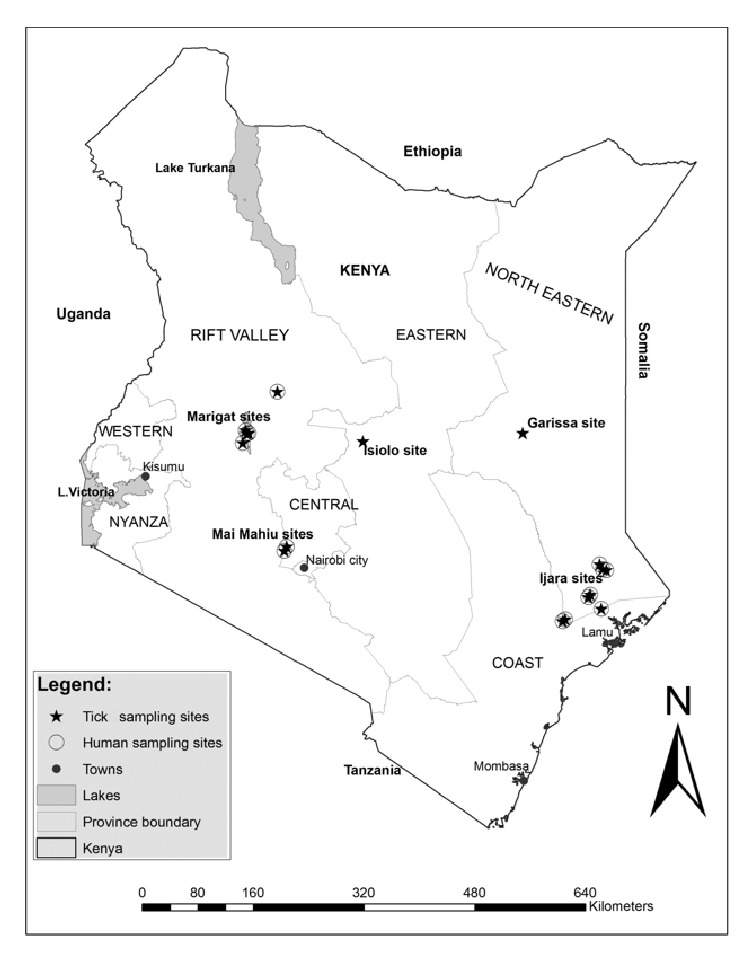
Map of sites where ticks and human samples had been collected.

This study was approved by the Institutional Review Boards at Kenya Medical Research Institute (KEMRI study # 2454) and the Walter Reed Army Institute of Research (WRAIR study #2099). Collection of human samples and ticks had been previously approved by the KEMRI IRB (Study #1560 and #824) and WRAIR IRB study #1134. The study set out to test for* Rickettsia* spp.,* Babesia* spp., and* Coxiella burnetii* in human samples and ticks from five pastoral communities (Figure 1) and a thesis was generated [24]. The results on* Rickettsia* spp. detected from this study were reported recently [25]. Herein, we present the results on detection of* Coxiella burnetii.*

### 2.2. Human Blood and Tick Samples

The human samples had been collected between December 2011 and December 2012 at dispensaries in three sites: Marigat, Mai Mahiu, and Ijara. Whole blood samples were collected from children >1 year of age and adults presenting at dispensaries with unexplained fever (>37.5°C) and other symptoms including diarrhoea, chills, muscle aches, joint pains, coughs, abdominal pain, and vomiting. Demographic data that had been collected from the study subjects included date of collection, village of residence, age in years, sex, occupation, tick bite, symptoms, clinician's diagnosis, and contact with animals.

The 380 tick pools tested in this study were collected from Marigat, Mai Mahiu, Ijara, Garissa, and Isiolo. Whole adult ticks had been identified to the species level using two tick identification keys. The ticks were then pooled in groups of 1 to 8 according to sex, developmental stage, species, area, site, collection date, and host. The tick pools were identified, homogenized, and processed as described by Koka* et al.,* 2017 [25].

### 2.3. DNA Extraction and Polymerase Chain Reaction Amplification

DNA was extracted from both tick and human samples using the Qiagen DNeasy Blood and Tissue kit (Qiagen Inc., Valencia, CA). The DNA was quantified using a Nanodrop 2000 spectrophotometer (Thermo Fisher scientific) and stored at -70 to -80°C.* Coxiella burnetii* was detected using a single step conventional PCR assay using the primers Trans 1 and Trans 2 [4] designed to amplify a 687-bp fragment of the repetitive, transposon-like IS1111 region. The PCR amplification conditions for the Trans primers included an initial denaturation step at 95°C for 2 min, followed by five cycles at 94°C for 30 s, 66 to 61°C (the temperature was decreased by 1°C between consecutive steps) for 1 min, and 72°C for 1 min. These cycles were followed by 35 cycles of 94°C for 30 s, 61°C for 30 s, and 72°C for 1 min and then a final extension step of 10 min at 72°C.* Coxiella burnetii* DNA was used as a positive control and water was used as a negative control. The PCR assays were performed in a Gene Amp 9700 thermocycler (Applied Biosystems) using a Taq PCR master mix kit (Qiagen Inc., Valencia, CA), 1ng of template DNA, and 1*μ*l of 50 *μ*M of the Trans primer in a 25*μ*l reaction mix. PCR products were separated on a 2% agarose gel visualized with ethidium bromide on a UV transilluminator. Products were sized using an O'rangeRuler 100bp DNA ladder (Thermo Fisher Scientific).

### 2.4. Sequencing and Data Analysis

Positive PCR products were purified and sequenced. The nucleotide sequences obtained in this study are available in GenBank under accession numbers: MG710507-MG710510. Sequencing and statistical analysis of the data were carried out as previously described by Koka et al. 2017 [25].

## 3. Results

### 3.1. Prevalence of* Coxiella burnetii* in Ticks

All the human blood samples were negative for* C. burnetii *DNA. On the contrary,* C. burnetii *was detected in 5.53% (95% CI 3.45-8.32) of the tick pools tested. The prevalence of* C. burnetii *varied significantly (p= 0.006) across the sites with Mai Mahiu recording the highest prevalence at 13.16%, followed by Marigat (7.89%), Isiolo and Ijara (both 2.63%), and Garissa recording only a single positive tick pool. The number of tick pools positive for* C. burnetii *was not significant with respect to animal host (p= 0.152): sheep (10.61%), goat (5.34%), and cattle (4.58%), and no tick pools from camels were positive.* C. burnetii *(Table 1) was predominantly detected in* Rhipicephalus* tick species (95.2%).

### 3.2. *Coxiella burnetii* Identified from the PCR Positive Tick Samples

Four of the twenty-one* C. burnetii* positive samples from ticks were sequenced and compared with sequences in GenBank. Two of the amplicons sequenced were derived from* Rh. evertsi evertsi* tick pools from Mai Mahiu, one from sheep and one from cattle. The other two amplicons were from* Rh. pulchellus* tick pools from Garissa and Isiolo, collected from a goat and cattle, respectively. All four* C. burnetii* positive tick samples were 94-97% homologous to the virulent strain CbCVIC1 and strain Heizberg from the Netherland outbreak.

## 4. Discussion

### 4.1. Prevalence of* Coxiella burnetii* in Tick Samples

*C. burnetii *was detected in a small percentage of ticks (5.5%) collected from sheep, goats, and cattle. The prevalence rate is comparable to the 6.4% reported in ticks collected from cattle in Ethiopia [26]. On the contrary, a study done in rural Western Kenya reported a lower prevalence of 2.5% in ticks collected from cattle [27] where zero grazing is commonly done. The slightly higher prevalence of* C. burnetii *in ticks in this study may be associated with the livestock rearing practice of grazing animals in the pastoral communities [11].

40 species of ticks are known to be infected with* C. burnetii *worldwide [1]*. Rh. evertsi evertsi, *the most widespread species of all the* Rhipicephalus* ticks in Africa (Walker 2003), was the predominant tick species infected with* C. burnetii* in this study. This is consistent with other studies in Senegal, Nigeria, and Kenya which detected* C. burnetii* DNA in* Rh. evertsi evertsi *tick species [18, 28, 29]. The detection of* C. burnetii* DNA in* Rh. appendiculatus* in this study corroborates previous reports of the same findings in Kenya [18, 27]. In addition,* C. burnetii *DNA was detected in* Rh. pulchellus* and* A. gemma* tick species.* C. burnetii* infection in these two tick species has been reported before in Ethiopia [26] while* C. burnetii* infection in* Rh. pulchellus* was recently reported in Kenya [18].

The ticks in our study were collected from sheep, goats, and cattle but these ticks also feed on donkeys, horses, and wild ungulates [30]. Ticks are able to transmit* C. burnetii* to these animals when they take a blood meal but some ticks are also able to transmit the infection transovarially [28]. We postulate that the ticks in our study may be involved in the transmission cycle of* C. burnetii* between domestic animals and wildlife. This is corroborated by the detection of* C. burnetii *from* Rh. evertsi evertsi, Rh. appendiculatus,* and* Rh. pulchellus* ticks collected from zebras and buffalos in Laikipia [18]. Importantly,* C. burnetii *DNA was not detected in ticks collected from camels in our study, despite a high* C. burnetii *seroprevalence being reported in camels in the region [31, 32].

The study also indicated a distinct geographical distribution of the positive ticks, with ticks from Mai Mahiu and Marigat being significantly more infected with* C. burnetii* than the other sites. Furthermore, the* C. burnetii* positive tick samples that were sequenced were closely related to the virulent strain CbCVIC1 and strain Heizberg associated with the largest outbreak of* C. burnetii* in the Netherland in 2007-2010 [33]. The sequenced samples were also related to the virulent Cb 175 epidemic strain from French Guyana known to cause the highest prevalence of community acquired pneumonia in the world [12]. Therefore, the animal and human populations in these two sites are at considerably higher risk of getting a Q fever infection. An outbreak of Q fever in humans had been reported in Baringo, by the Zoonotic Disease Unit in Kenya in March 2014 [34]. However, in this study* C. burnetii* DNA, the causative agent of Q fever, was not detected in the human blood samples tested. This is in spite of activities such as herding, slaughtering of cattle, and milking that allow contact with infected animals which are carried out in these communities [35].

A limitation of this study was the lack of human samples from Garissa and Isiolo which prevented direct correlation of human and tick infections. No human samples were collected from these sites in the previous studies from which the archived samples were obtained. Another limitation of this study was the time difference in sampling humans and ticks in these sites making it challenging to infer tick to human transmission. A distinct seasonality of Q fever related to the parturient season in domestic and wild animal has been established [36]. Therefore, it is likely that samples collected in the parturient season would provide different results.

## 5. Conclusion

The study identified* Rh. evertsi evertsi *and* Rh. pulchellus* as the tick species predominantly infected with* C. burnetii.* The findings in this study suggest that grazing of cattle, sheep, and goats in the pastoral communities increases the risk of acquiring* C. burnetii* infection from ticks. We suggest that a robust surveillance system in domestic animals and humans be established to monitor this disease in these two locations. More emphasis should also be placed on acaricide treatment of all livestock to prevent the spread of tick-borne infection.

## Figures and Tables

**Table 1:** Tick species collected from livestock and the tick species positive for *Coxiella burnetii*.

**Tick species**	**# ticks per animal host**					***Coxiella PCR positive pools***	
	**Camel**	**Cattle**	**Goat**	**Sheep**	**Total**	**n**	%
*Amblyomma gemma*	1	11	4	1	**17**	1	5.9
*Amblyomma hebraeum*	0	0	1	0	**1**	0	0.0
*Amblyomma lepidum*	0	3	4	0	**7**	0	0.0
*Amblyomma variegatum*	0	1	2	0	**3**	0	0.0
*Amblyomma spp*	0	1	0	0	**1**	0	0.0
*Rhipicephalus annulatus*	0	0	0	1	**1**	0	0.0
*Hyalomma marginatum*	1	13	1	0	**15**	0	0.0
*Hyalomma truncatum*	7	14	4	4	**29**	0	0.0
*Hyalomma spp*	6	2	0	0	**8**	0	0.0
*Rhipicephalus appendiculatus*	0	4	17	8	**29**	2	6.9
*Rhipicephalus pulchellus*	15	79	79	28	**201**	8	4.0
*Rhipicephalus evertsi evertsi*	0	25	19	24	**68**	10	14.7
**Total **	**30**	**153**	**131**	**66**	**380**	**21**	
**Coxiella PCR positive pools (n) **	0	7	7	7	**21**		
**Coxiella PCR positive pools (%)**	0	4.6	5.3	10.6			

## Data Availability

The data used to support the findings of this study are available from the corresponding author upon request.
